# The effect of d-cycloserine on brain processing of breathlessness over pulmonary rehabilitation: an experimental medicine study

**DOI:** 10.1183/23120541.00479-2022

**Published:** 2023-04-03

**Authors:** Sarah L. Finnegan, Olivia K. Harrison, Sara Booth, Andrea Dennis, Martyn Ezra, Catherine J. Harmer, Mari Herigstad, Bryan Guillaume, Thomas E. Nichols, Najib M. Rahman, Andrea Reinecke, Olivier Renaud, Kyle T.S. Pattinson

**Affiliations:** 1 Nuffield Department of Clinical Neurosciences, University of Oxford, Oxford, UK; 2 Translational Neuromodeling Unit, Institute for Biomedical Engineering, University of Zurich and ETH Zurich, Zurich, Switzerland; 3 Department of Psychology, University of Otago, Dunedin, New Zealand; 4 Cambridge Breathlessness Intervention Service, CUNHSFT, Cambridge, UK; 5 Perspectum Ltd, Oxford, UK; 6 Department of Psychiatry, Medical Sciences, University of Oxford, Oxford, UK; 7 Oxford Health NHS Foundation Trust, Warneford Hospital Oxford, Oxford, UK; 8 Department of Biosciences and Chemistry, Sheffield Hallam University, Sheffield, UK; 9 Oxford Big Data Institute, Li Ka Shing Centre for Health Information and Discovery, Nuffield Department of Population Health, University of Oxford, Oxford, UK; 10 Nuffield Department of Medicine, University of Oxford, Oxford, UK; 11 Oxford NIHR Biomedical Research Centre, Oxford, UK; 12 Oxford Chinese Academy of Medicine Institute, Oxford, UK; 13 Department of Psychology, University of Geneva, Geneva, Switzerland; 14 Nuffield Department of Anaesthetics, Oxford University Hospitals NHS Foundation Trust, Oxford, UK

## Abstract

**Research question:**

Pulmonary rehabilitation is the best treatment for chronic breathlessness in COPD but there remains an unmet need to improve efficacy. Pulmonary rehabilitation has strong parallels with exposure-based cognitive behavioural therapies (CBT), both clinically and in terms of brain activity patterns. The partial N-methyl-d-aspartate (NMDA)-receptor agonist d-cycloserine has shown promising results in enhancing efficacy of CBT, thus we hypothesised that it would similarly augment the effects of pulmonary rehabilitation in the brain. Positive findings would support further development in phase 3 clinical trials.

**Methods:**

72 participants with mild-to-moderate COPD were recruited to a double-blind pre-registered (ClinicalTrials.gov identifier: NCT01985750) experimental medicine study running parallel to a pulmonary rehabilitation course. Participants were randomised to 250 mg d-cycloserine or placebo, administered immediately prior to the first four sessions of pulmonary rehabilitation. Primary outcome measures were differences between d-cycloserine and placebo in brain activity in the anterior insula, posterior insula, anterior cingulate cortices, amygdala and hippocampus following completion of pulmonary rehabilitation. Secondary outcomes included the same measures at an intermediate time point and voxel-wise difference across wider brain regions. An exploratory analysis determined the interaction with breathlessness anxiety.

**Results:**

No difference between d-cycloserine and placebo groups was observed across the primary or secondary outcome measures. d-cycloserine was shown instead to interact with changes in breathlessness anxiety to dampen reactivity to breathlessness cues. Questionnaire and measures of respiratory function showed no group difference. This is the first study testing brain-active drugs in pulmonary rehabilitation. Rigorous trial methodology and validated surrogate end-points maximised statistical power.

**Conclusion:**

Although increasing evidence supports therapeutic modulation of NMDA pathways to treat symptoms, we conclude that a phase 3 clinical trial of d-cycloserine would not be worthwhile.

## Abstract 2

**Although increasing evidence supports therapeutic modulation of NMDA pathways to treat symptoms of breathlessness in COPD, d-cycloserine does not demonstrate sufficient efficacy to be considered for phase 3 trials in pulmonary rehabilitation** https://bit.ly/3Wm64ni

## Introduction

Chronic breathlessness is a central symptom of COPD. Currently, pulmonary rehabilitation offers the most effective treatment strategy for chronic breathlessness in COPD. However, around 30% of patients derive no clinical benefit [1]. Health-related benefits plateau within the first 6 months following pulmonary rehabilitation, returning to pre-rehabilitation levels for the majority of patients after 12–18 months [2]. Thus, there remains an unmet need to develop strategies to increase or prolong the beneficial effects of pulmonary rehabilitation.

A body of evidence has shown that improvements in breathlessness over pulmonary rehabilitation result from a reappraisal of the sensory experience [3, 4], arresting the downward spiral of fear, avoidance and physical deconditioning. The safe, graded exposure to breathlessness within pulmonary rehabilitation parallels techniques of exposure-based cognitive behavioural therapy (CBT), in which pathological fears are first activated and then disconfirmed by new adaptive information. Although these parallels are drawn observationally, practitioners in CBT are explicitly trained in graded exposure theories, while pulmonary rehabilitation physiotherapists are not; clinical studies of both pulmonary rehabilitation and CBT have shown changes to brain activity within areas associated with attention and learned sensory and emotional expectations such as the cingulate cortex, angular gyrus, insula and supramarginal gyrus [4, 5].

In the field of psychiatry, there has been great interest in the partial N-methyl-d-aspartate (NMDA) agonist d-cycloserine as a pharmacological adjunct to enhance efficacy of exposure-based CBT [6–8]. d-cycloserine is thought to act at the glycine modulatory site of the NMDA receptor. Its high affinity binding enhances synaptic plasticity, promoting emotional learning processes [8] boosting therapeutic effects of CBT as a result [7, 9, 10]. Experimental medicine studies have demonstrated reductions in emotional response within the amygdala when paired with CBT [6], increasing activity within the hippocampus in a manner linked to learning [11]. In CBT for alcoholism, d-cycloserine decreased cue-induced brain activity across the ventral and dorsal striatum, which was associated with reductions in alcohol craving [12]. Clinical trials of d-cycloserine paired with CBT have demonstrated reductions in symptoms of acrophobia [13], social phobia [14], panic disorder [15] and obsessive compulsive disorder over placebo [16, 17], often with medium to large effect sizes.

Given the strong parallels between pulmonary rehabilitation and exposure-based CBT, and the strong effects of pulmonary rehabilitation on affective components of breathlessness [3, 4], we hypothesised that d-cycloserine may have therapeutic benefits in enhancing pulmonary rehabilitation.

To test this hypothesis, we performed an experimental medicine study using functional magnetic resonance imaging (FMRI) markers of drug efficacy in the brain. We chose this approach over a phase 3 clinical trial because the differences between COPD populations and those with primary psychiatric conditions in which d-cycloserine has been tested so far might necessitate a bespoke trial design. Therefore, we wanted to test efficacy in the brain first. A positive result from an FMRI study would facilitate clinical trial design and help early decisions of go/no-go on further clinical development and subsequent larger phase 3 clinical trials.

Since the inception of this study, there has been an increasing number of null results from studies using d-cycloserine in combination with exposure-based CBT [16, 18]. While this trend may in part be explained by technical differences in study design, a number of well-powered and well-controlled studies have also revealed a more nuanced picture of d-cycloserine action. Hofmann *et al.* [17] suggest that “d-cycloserine not only makes ‘good exposures’ better but also may make ‘bad exposures’ worse”. To account for this updated literature, we conducted an additional analysis, which tested whether brain activity changes relating to d-cycloserine was linked to improvements in breathlessness-related anxiety during pulmonary rehabilitation.

## Methods and materials

An overview of the methodology is presented here. Full details, including non-completion and sensitivity analysis can be found within the supplementary material. The study and statistical analysis plan were pre-registered on clinicaltrials.gov (ID: NCT01985750) prior to unblinding. This is the first example of pre-registration of both study design and analysis plan in a respiratory neuroimaging study.

### Sample size

At the time of study inception (and to a large extent still to date), the literature regarding d-cycloserine's effects on functional brain activity is very limited. Therefore, in order to calculate the sample sizes required for this study we first took into account the described effects of d-cycloserine in clinical studies of augmentation for CBT for anxiety disorders, where effect sizes of up to 1.06 have been reported (although more commonly 0.4 to 0.7) [13, 14, 19]. The most relevant paper (on treatment of snake phobia [10]) demonstrated that effects observed with neuroimaging were more sensitive than behavioural effects; therefore powering for a behavioural outcome measure (breathlessness anxiety) provided a safe margin and was likely to be sufficiently conservative to detect our measures of interest. This was particularly the case as compared to the relatively blunt nature of behavioural data collection, functional neuroimaging carries considerably more specificity and statistical power. The study was not therefore specifically powered to investigate the clinical effects of d-cycloserine. In our previous study we observed a mean±sd improvement in breathlessness-related anxiety of 11±15%, measured with our FMRI word task (pre-treatment mean score 38%, post-treatment mean score 27%, difference 11%, sd of difference 15%) [4]. Making a conservative assumption, we estimated that d-cycloserine augments this response with an effect size of 0.4. Assuming a similar coefficient of variation we anticipated a mean±sd improvement in breathlessness anxiety of 18±24% (*i.e.* pre-treatment mean score 38%, post treatment mean score 20%, difference 18%, sd of difference 24%). Assuming α=0.05 and power 0.80, then we estimated a sample size of 36 in each group randomised 1:1. As this is a behavioural outcome, we expected this to have sufficient power to detect change in blood oxygen level-dependent signalling.

### Participants

**FIGURE 1:**
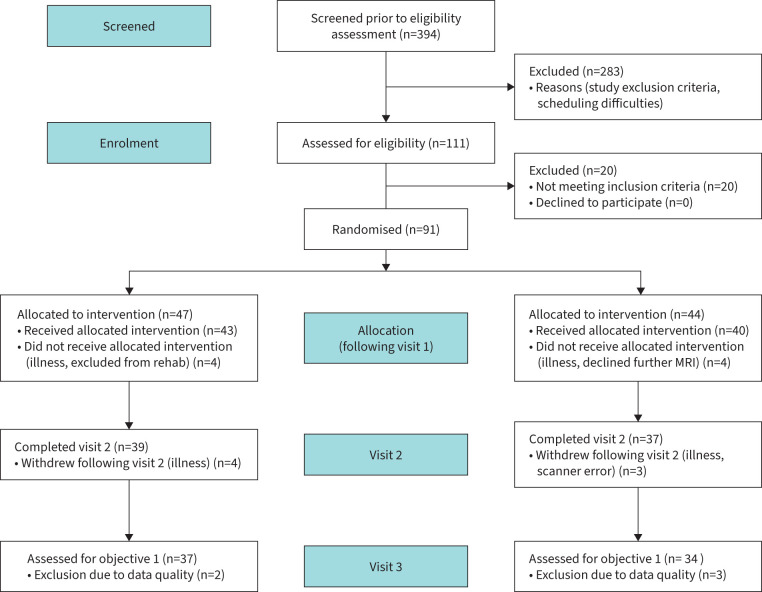
Consort diagram illustrating stages of participant recruitment from initial screening through to completion at visit 3. MRI: magnetic resonance imaging.

91 participants (30 female, median age 70 years; range 46–85 years) with COPD were recruited immediately prior to their enrolment in a UK National Health Service-prescribed course of pulmonary rehabilitation (full demographic information including non-continuation is shown in supplementary table S1). From this population, 72 participants completed all study visits (18 female, median age 71 years (46–85 years)) (table 1 and figure 1). Written informed consent was obtained from all participants prior to the start of the study. Study approval was granted by South Central Oxford REC B (Ref: 118784, Ethics number: 12/SC/0713). Study inclusion criteria were: a diagnosis of COPD and admittance to pulmonary rehabilitation. Exclusion criteria were: inadequate understanding of verbal and written English, significant cardiac, psychiatric (including depression under tertiary care) or metabolic disease (including insulin-controlled diabetes), stroke, contraindications to either d-cycloserine (including alcoholism) or magnetic resonance imaging (MRI), epilepsy, claustrophobia, regular therapy with opioid analgesics or home oxygen therapy.

**TABLE 1:** Demographic information from the 72 participants who completed all three study visits

	**Visit 1 (pre-rehabilitation)**	
	**d-cycloserine**	**Placebo**
**Age years, median (range)**	71.0 (47–81)	71.5 (46–85)
**Smoking pack-years, median (IQR)**	34.0 (25.6)	30.0 (30.0)
**Duration of breathlessness years, median (IQR)**	8.0 (17.0)	9.5 (10.0)
**Total exacerbations, median (IQR)**	0.0 (1.0)	1.0 (2.3)
**BMI kg·m^−2^, mean±sd**	27.3±6.5	26.9±5.7
**MRC breathlessness scale, median (IQR)**	3.0 (1)	2.5 (1)
**Resting *S***_**pO_2_**_ **%, median (IQR)**	95 (3.3)	95 (3.0)
**Resting heart rate beats·min^−1^, mean±sd**	80.8±13.4	80.8±14.9
**FEV_1_/FVC, median (IQR)**	0.53 (0.17)	0.56 (0.13)
**FEV_1_ % predicted, median (IQR)**	51.7 (31.2)	66.8 (25.2)
**GOLD n**		
1 (A/B/C/D)	10 (0/10/0/0)	6 (1/5/0/0)
2 (A/B/C/D)	14 (3/11/0/0)	14 (0/14/0/0)
3 (A/B/C/D)	12 (0/0/0/12)	11 (0/0/0/11)
4 (A/B/C/D)	1 (0/0/0/1)	3 (0/0/0/3)
**Comorbidities n**		
** **Asthma	14	11
** **Hypertension	13	11
** **Gastro-oesophageal reflux	10	12
** **Swelling of both ankles	11	8
** **Surgery to the chest	6	7
** **Depression	6	2
** **Diabetes	3	6
** **Heart attack	4	5
** **Bronchiectasis	3	4
** **Osteoporosis	2	4
** **Arrhythmia	3	4
** **Inflammatory bowel disease	2	3
** **Peptic ulcer	3	2
** **Heart failure	1	1
** **Tuberculosis	1	2

Variance is expression either in terms of sd or interquartile range (IQR) depending on the normality of the underlying data distribution. BMI: body mass index; MRC: Medical Research Council; *S*_pO_2__ %: peripheral oxygen saturation, expressed as a percentage; FEV_1_: forced expiratory volume in 1 s; FVC: forced vital capacity; GOLD: Global Initiative for Chronic Obstructive Lung Disease.

### Study drug

Participants were randomised in a double-blinded procedure to receive either 250 mg oral d-cycloserine or a matched placebo, administered by the study nurse 30 min prior to the onset of their first four pulmonary rehabilitation sessions. 250 mg dosage has been shown to be efficacious [6, 11]. d-cycloserine exerts its best effects when given immediately before exposure-based therapy and only for a limited number of times [20] with daily administration associated with tachyphylaxis [13]. Therefore, the dose timing of d-cycloserine was deliberately selected to be the first four sessions, where the greatest potential for emotional learning occurs as patients become habituated to pulmonary rehabilitation. Study participants, investigators and those performing the analysis were blinded to the treatment allocation. Both d-cycloserine and placebo were over-encapsulated to appear identical.

The following minimisation criteria were used for randomisation: centre at which pulmonary rehabilitation was performed, Medical Research Council (MRC) breathlessness grade, presence of diabetes, whether the participant was taking antidepressants, the age at which the participant completed full time education and previous pulmonary rehabilitation. Full details of randomisation can be found within the supplementary material.

### Study visit protocol

**FIGURE 2:**
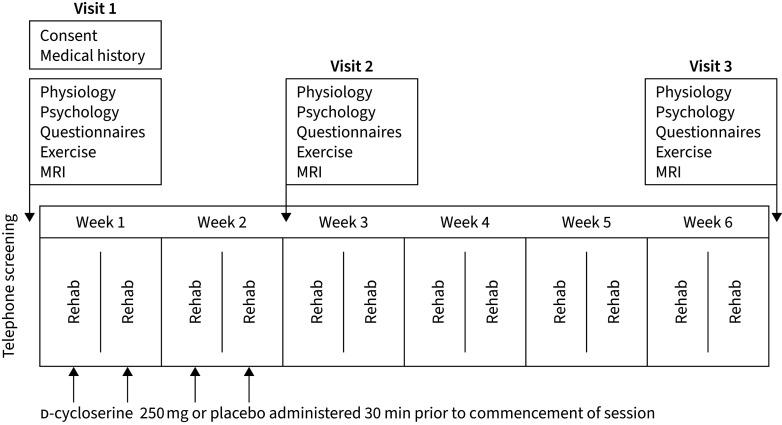
A schematic demonstrating order of visits, rehabilitation sessions and tablet administration throughout the study period. Participants took part in one study visit prior to their first pulmonary rehabilitation session. Study drug/placebo was administered on four occasions over the first four rehabilitation sessions. A second study visit occurred after the final drug/placebo administration. Participants continued with their pulmonary rehabilitation course for a further 4 weeks before returning for a third study visit. MRI: magnetic resonance imaging.

Following telephone screening, participants were invited to attend their first research session (baseline) prior to starting pulmonary rehabilitation. A second study visit took place following the fourth pulmonary rehabilitation session but before the sixth session. Participants completed the remainder of their pulmonary rehabilitation course before attending a third study session (figure 2) that occurred as close to the termination of pulmonary rehabilitation as possible and always within 2 weeks.

### Pulmonary rehabilitation

Pulmonary rehabilitation courses were run by either Oxford Health NHS Foundation Trust, West Berkshire NHS Foundation Trust, or Milton Keynes University Hospitals NHS Trust. The full course ran for 6 weeks with two sessions per week including an hour of exercises and an hour of education, as part of a standard pulmonary rehabilitation programme. For programme details see the supplementary material.

### Self-report questionnaires

Building on our previous work [4, 21] we selected a set of questionnaires with proven sensitivity to changes across pulmonary rehabilitation [4], which were designed to probe the experience of living with COPD. These were scored according to their respective manuals: Dyspnoea-12 (D12) Questionnaire [22], Centre for Epidemiologic Studies Depression Scale (CES-D) [23], Trait Anxiety Inventory (TRAIT) [24], Fatigue Severity Scale [25], St George's Respiratory Questionnaire (SGRQ) [26], MRC breathlessness scale [27], Mobility Inventory (MI) [28], Breathlessness Catastrophising Scale, adapted from the Catastrophic Thinking Scale in Asthma [29], Breathlessness Vigilance, adapted from the Pain Awareness and Vigilance Scale [30].

### Physiological measures

Spirometry and two modified shuttle walk tests (MSWT) were collected using standard protocols [31, 32]. Participant height and weight were recorded at each visit. Oxygen saturations and heart rate were measured with pulse oximetry and were collected at rest and following the MSWT.

### MRI measures

#### Image acquisition

MRI of the brain was carried out using a Siemens (Munich, Germany) 3T MAGNETOM Trio. A T1-weighted (MPRAGE) structural scan (voxel size: 1×1×1 mm) was collected and used for registration purposes. A T2*-weighted, gradient echo planar image (EPI) scan sequence (voxel size: 3×3×3 mm; TR 3000 ms, TE 30 ms) was used to collect FMRI data.

#### Word cue task

To probe the neural responses of breathlessness-related expectations we examined the activity of brain regions responding to breathlessness-related word cues [4, 21, 33]. This paradigm has previously been shown to be sensitive to improvements in breathlessness over a course of pulmonary rehabilitation. Brain activity was correlated with corresponding visual analogue ratings of breathlessness and breathlessness anxiety collected during scanning [4]. During FMRI scanning, participants were presented with a word cue, *e.g.*, “climbing stairs” in white text on a black background for 7 s. Participants were then asked, “how breathless would this make you feel” (wB) and “how anxious would this make you feel” (wA). Participants responded to each question within a 7-s window using a button box and visual analogue scale. The response marker always initially appeared at the centre of the scale, with the anchors “Not at all” and “Very much” at either end. Scan duration was 7 min and 33 s.

#### Control task

A validated task of emotional faces was used as a control to separate generalised anxiety from breathlessness-specific anxiety. Fearful or happy faces presented on a black background was used to examine whether any differences in brain activity patterns between d-cycloserine and placebo groups was specific to breathlessness processing. Each face was shown for 500 ms in blocks of 30 s. A fixation cross was interspersed for 30 s between the blocks of faces. Participants were instructed to respond *via* a button box to indicate facial gender. Reaction time and accuracy were recorded throughout the task. Scan duration was 5 min and 42 s.

### Outcomes

Our primary outcomes focused on brain activity changes within five key regions of interest, identified in previous studies of breathlessness [4, 34]. The regions of the anterior insular cortex, posterior insular cortex, anterior cingulate cortex, amygdala and hippocampus have all been linked to body and symptom perception as well as emotional salience [35, 36]. Focusing on a small number of regions of interest is more statistically powerful and therefore more likely to detect a hypothesised difference. Secondary hypotheses examined the effect of d-cycloserine across a wider region of interest containing 15 pre-defined brain areas. The 15 brain areas encompassed regions associated with sensory and affective processing of breathlessness as well as body and symptom perception regions. Exploratory analyses updated the models used for primary and secondary outcomes to incorporate psychological variables.

### Analysis

A summary of analyses is outlined here. Full details, including procedures for dealing with missing data and sensitivity analysis, can be found within the supplementary material. Our primary and secondary analysis was pre-registered and made publicly available prior to unblinding https://mfr.osf.io/render?url=https://osf.io/wqyf4/?action=download%26mode=render.

#### Brain imaging analysis

Image processing was carried out using the Oxford Centre for Functional Magnetic Resonance Imaging of Brain Software Library (FMRIB, Oxford, UK; FSL version 5.0.8; https://fsl.fmrib.ox.ac.uk/fsl/fslwiki/FSL), MATLAB R2018b (Mathworks, Natick, MA, USA), R-studio, R version 3.6.1 (2019-07-05) and associated custom scripts. Functional MRI processing was performed using FEAT (FMRI Expert Analysis Tool, within the FSL package).

Data were pre-processed according to standard protocols before being entered into single subject general linear models. These models captured brain activity during the periods in which the breathlessness-related word cues were presented allowing us to examine expectation-related processes.

#### Group level analysis

For each patient, the following metrics were extracted from each of the five regions of interest: anterior insula cortex, posterior insula cortex, anterior cingulate cortex, amygdala and hippocampus, at visits one, two and three:

1. mean brain activity in response to breathlessness word cue presentation
2. mean brain activity for control task of emotional faces

Baseline group level brain activity in response to breathlessness-related word cues can be seen in supplementary figure 7 in Finnegan *et al.* [37] in which activity was observed within inferior frontal gyrus, posterior cingulate, anterior cingulate and insula. To test for a drug effect across each metric, the values from visit two were entered into independent linear mixed effects models where they were adjusted for age, sex and scores at visit one. To correct for multiple comparisons across regions, permutation testing (with family-wise error rate 5%) was carried out. This process was repeated separately for data collected at visit three. Models were programmed using the lme4 function and permuco package within R version 3.6.1 (2019-07-05).

To test for a drug effect across the larger region of interest (panel B of supplementary figure S1), the following voxel-wise information was collected from within the region of interest at visits one, two and three:

1. voxel-wise brain activity in response to breathlessness word-cues presentation
2. voxel-wise brain activity in response to the control task of emotional faces

Each of the values from visit two were entered into the independent general linear model, controlling for age, sex and scores at visit one. Permutation testing was performed with threshold-free cluster enhancement (a non-parametric test) [38] using FSL's Randomise tool [39] at family-wise error rate corrected, p<0.05. The process was then repeated separately for data collected at visit three.

We then repeated the primary and secondary analysis but with an additional term included in each model for the change in breathlessness-related anxiety (wA). This allowed us to ask the question “Was there a difference in the relationship of brain activity and changes in breathlessness anxiety between the d-cycloserine and placebo groups?”.

## Results

Of the 91 participants recruited (figure 1), 72 participants completed all three study visits. Reasons for dropout or exclusion included illness, scanner error and issues with data quality. One further participant was excluded due to an error in task-data collection. 71 participants were therefore assessed for study objectives. Sensitivity analysis was performed and is reported within the supplementary material.

### Primary outcomes

There was no significant overall effect of d-cycloserine on mean brain activity within the five key regions of interest of anterior cingulate, anterior insula cortex, amygdala, hippocampus or posterior insula cortex (family-wise error rate corrected, p>0.05) at visit two or visit three (table 2).

**TABLE 2:** Significance of overall effect of d-cycloserine on mean brain activity within the five key regions of interest at visits two and three, having accounted for scores at visit one

**Region of interest**	**Estimate**	_SE_	**p-value**
**Visit two**			
*** ***Anterior cingulate cortex	0.066	0.059	0.70
** **Anterior insula	0.019	0.058	0.98
** **Amygdala	0.097	0.059	0.40
** **Hippocampus	0.018	0.058	0.98
** **Posterior insula	−0.013	0.059	0.98
**Visit three**			
** **Anterior cingulate cortex	−0.009	0.056	0.98
** **Anterior insula	0.019	0.055	0.98
** **Amygdala	0.055	0.056	0.78
** **Hippocampus	0.014	0.056	0.98
** **Posterior insula	0.068	0.056	0.70

Significance is reported as family-wise error (p<0.05) corrected p-values of the difference.

### Secondary outcomes

There was no significant overall effect of d-cycloserine across the broader mask of 15 regions measured voxel-wise (family-wise error rate corrected, p>0.05) at visit two or visit three.

No significant differences in the questionnaire measures or physiology scores were found between the d-cycloserine and placebo groups at any point during the study. No differences were observed between the two groups either in breathlessness ratings (wB) or breathlessness-related anxiety (wA) at visit two (both p=0.15 family-wise error rate corrected) (supplementary table S9) or visit three (both p=0.053 family-wise error rate corrected) (supplementary table S10). Group level effects of pulmonary rehabilitation on brain activity in response to breathlessness-related word cues can be observed in supplementary figure S3. No significant effect of drug group was identified, using repeat measured ANOVAs, for the emotional faces control task (collected during FMRI scanning) at visit two (F(1,68)=0.17, p=0.68) or visit three (F(1,68)=0.001, p=0.97). Furthermore, no significant interaction between drug group and emotional valence (happy or fearful faces) was identified at visit two (F(1,68)=0.36, p=0.55) (supplementary table S9) or visit three (F(1,68)=0.002, p=0.97) (supplementary table S10). Raw scores are reported in supplementary table S4.

Overall changes in self-report questionnaires over the course of pulmonary rehabilitation were as expected and are presented in supplementary table S5.

### Exploratory outcomes

**FIGURE 3:**
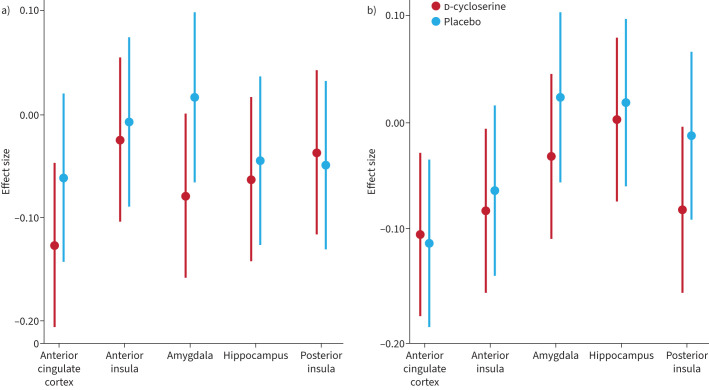
Effect sizes and confidence intervals for each of the five regions of interest at visits a) two and b) three for d-cycloserine and placebo groups.

**FIGURE 4:**
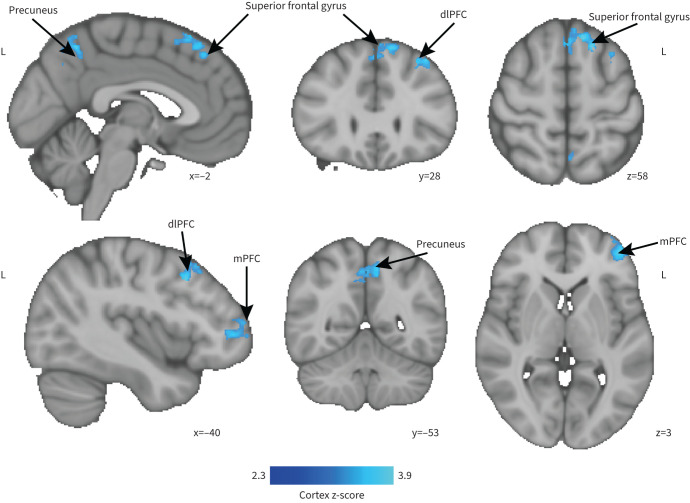
Changes in blood oxygen level-dependent (BOLD) activity that correlate with changes in ratings of breathlessness-related anxiety (wA). Changes in BOLD activity are shown here for placebo group as greater than d-cycloserine group during the presentation of breathlessness-related word cues (cluster corrected threshold of z=2.3, p<0.05). dlPFC: dorsolateral prefrontal cortex; mPFC: medial prefrontal cortex/frontal pole.

**FIGURE 5:**
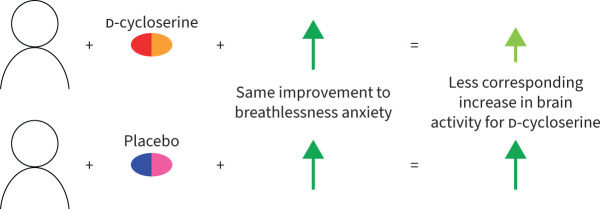
Pictorial representation of the relationship between drug allocation (d-cycloserine/placebo), changes to breathlessness anxiety and brain activity shown in figure 4.

Following completion of the pulmonary rehabilitation course (visit three) we observed an interaction between changes in breathlessness anxiety, drug allocation (d-cycloserine/placebo) and brain activity (figure 3). In the d-cycloserine group compared to the placebo group, for a given improvement in breathlessness anxiety there was an attenuated neural response to breathlessness cues (z=2.3, p<0.05) (figure 4). This difference in brain activity was observed within the dorsolateral and medial prefrontal cortices, superior frontal gyrus and precuneus. A pictorial representation of this relationship can be seen in figure 5. No significant relationship was observed between breathlessness-related anxiety, drug allocation and brain activity at visit two.

## Discussion

### Key findings

We found that 250 mg of d-cycloserine administered prior to the first four sessions of pulmonary rehabilitation showed no mean effect on breathlessness-related brain activity in the five regions of interest tested: amygdala, anterior insula, posterior insula, anterior cingulate cortex or hippocampus when assessed either during (after four to five sessions) or after the course. Likewise, there was no mean effect on the secondary end-points of voxel-wise activity across a wider selection of brain regions. These findings suggest that d-cycloserine does not have the potential to move to phase 3 clinical trials in pulmonary rehabilitation and that alternative drug candidates should be considered. However, the results from the exploratory analysis give important insights into potential mechanisms of action for brain-targeted drugs like d-cycloserine. In the d-cycloserine group, for a given change in ratings of breathlessness anxiety, there was correspondingly less brain activity in response to breathlessness cues than the placebo group. This effect was observed across a network of emotional salience regions, which included superior frontal gyrus, precuneus, dorsolateral prefrontal cortex and medial prefrontal cortex. This suggests a downregulation of emotional expectation-related responses to breathlessness word cues as a result of d-cycloserine in people who had derived positive change from pulmonary rehabilitation.

This is the first study to test a neuro-pharmacological adjunct to pulmonary rehabilitation. A key strength of this study, in addition to its sample size, which is large for a neuroimaging study of breathlessness, is its use of robust clinical trial methodology which was monitored in accordance with Good Clinical Practice standards. We included a formal sample size calculation powered for validated end-points relevant to the patient population, pre-registered the study design and published a statistical analysis plan ahead of unblinding. Additionally, our hypotheses focused on five *a priori* defined brain regions with demonstrated evidence of strong modulation by d-cycloserine [9, 40] or links to body and symptom perception as well as emotional salience [35, 36]. Together these steps, which are rarely carried out for neuroimaging studies, ensured rigorous methodology, robust findings and provide the best chance to detect an effect if present.

### Why was no drug effect observed across the group?

#### d-cycloserine may not have sufficient glutamatergic activity

While d-cycloserine has not shown sufficient promise to be progressed to full-scale clinical trials, other drugs may now be more attractive candidates. Paired with cognitive therapies as treatment for treatment-resistant depression, ketamine and its derivative esketamine, which blocks pre-synaptic NMDA-receptor signalling, increasing glutamate and thereby synaptic plasticity, has been linked with reductions in fear and anxiety, and rapid relief from symptoms [41].

#### Individual differences

Recent literature has highlighted the importance of individual differences in response to d-cycloserine. New evidence suggests that d-cycloserine has the potential to reinforce either positive or negative experiences during exposure-based CBT [17]. Direction of action appears to depend on stress levels, which at the neurochemical level influence neurotransmitter concentrations surrounding the NMDA receptors [42]. These opposing effects may render brain activity differences unobservable in a simple contrast of means. These effects may be further compounded by the multidimensional nature of breathlessness and corresponding heterogeneous symptoms of people living with COPD [37, 43, 44]. Our exploratory analysis investigated this further. Following the completion of pulmonary rehabilitation, for a given improvement in breathlessness anxiety, d-cycloserine suppressed brain activity in response to breathlessness-related word cues within the superior frontal gyrus, precuneus, dorsolateral prefrontal cortex and medial prefrontal cortex compared to placebo. This interaction can be considered as a difference in slopes of the relationship between breathlessness anxiety and brain activity.

The networks targeted by d-cycloserine are associated with attentional regulation. In a previous study these networks were also shown to change over pulmonary rehabilitation [4]. However, while our previous work found co-activation within angular and supramarginal gyrus, regions associated with somatosensory integration, the current study's co-activated networks are associated with emotional responsiveness. This finding demonstrated parallels between an earlier comparison of brain activity between patients with COPD and healthy controls, where breathlessness-related word cues elicited greater activity within medial prefrontal cortex than patients with COPD [21]. This difference was thought to reflect differences in emotional-cognitive aspects of breathlessness processing. Therefore, d-cycloserine, which as we have shown here modulates the medial prefrontal cortex, may be driving activity towards that seen in healthy controls for whom the breathlessness words hold less expectation-related significance. We speculate that our findings represent a downregulation of emotional responses to the breathlessness word cues as a result of d-cycloserine for people who had derived positive change (measured by breathlessness anxiety ratings) from pulmonary rehabilitation.

#### Ceiling effect

The action of d-cycloserine is known to be curtailed near the therapeutic ceiling [45], and pulmonary rehabilitation is a highly effective treatment [2]; this may leave insufficient scope for improvement in some individuals.

#### Little evidence of d-cycloserine in older adults

Older adults are not well represented within the evidence base regarding d-cycloserine's action. Given the well-established changes to NMDA-receptor function as the brain ages, d-cycloserine may act differently in this population [46].

#### Alternative brain pathways for pharmacological targets

In this study we specifically investigated drug effect on breathlessness-related brain activity. However, other targets may also positively impact breathlessness or pulmonary rehabilitation outcomes *via* other mechanisms. For example, mindfulness-based CBT in pulmonary rehabilitation can improve quality of life without affecting breathlessness [47]. Glucocorticoids such as cortisol, combined with exposure-based CBT, have shown promise in reduction of fear in phobias and post-traumatic stress disorder [7] *via* their action on glucocorticoid receptors along the hypothalamic–pituitary–adrenal axis. Findings regarding selective serotonin reuptake inhibitors (SSRIs) meanwhile are mixed. Paired with CBT, paroxetine was found to reduce panic attacks by 50% compared to placebo [48]. However, a review of wider SSRI literature found that chronic and sub-chronic administration was associated with reduced CBT response, leading to questions as to whether SSRIs may even interfere with CBT effectiveness [49]. Collectively these candidate drugs boost synaptic plasticity, although *via* different neurochemical pathways, which may facilitate the re-setting of fearful associations within the brain. These could be used either during pulmonary rehabilitation, or as part of a precursor programme, helping to recruit harder to reach patients and support self-management.

#### Dosing and dose timing

Questions still remain regarding d-cycloserine's optimum dosage, dose timing and number of administered sessions [16]. Based on the available literature at the time [6, 8] and practical considerations regarding drug availability, we selected a dose of 250 mg for this study, administered at the first four rehabilitation sessions. However, given that our maximum effect size was 0.18 (figure 3), even a 50% increase would be below the commonly reported effect sizes of 0.4–0.7, and up to 1.06 [10, 13, 14, 19]. This strongly suggests that dose and dose timing did not drive the negative result.

### Conclusions

We have shown evidence that d-cycloserine does not have a mean effect on breathlessness-related brain activity, behavioural or physiological measures over the course of pulmonary rehabilitation. Instead, the drug appears to work in a more nuanced manner and interacts with changes in breathlessness anxiety to influence the brain's breathlessness perception networks, lending support to personalised approaches to treating breathlessness. This study contributes important information regarding the overall (un)suitability of d-cycloserine as a candidate for phase 3 clinical trials in pulmonary rehabilitation.

## Supplementary material

10.1183/23120541.00479-2022.Supp1 **Please note:** supplementary material is not edited by the Editorial Office, and is uploaded as it has been supplied by the author.
Supplementary material 00479-2022.supplement
